# DPPC-coated lipid nanoparticles as an inhalable carrier for accumulation of resveratrol in the pulmonary vasculature, a new strategy for pulmonary arterial hypertension treatment

**DOI:** 10.1080/10717544.2020.1760962

**Published:** 2020-05-13

**Authors:** Zerong Li, Wenmei Qiao, Chenghao Wang, Heqiao Wang, Mengchao Ma, Xinyu Han, Jingling Tang

**Affiliations:** Department of Pharmaceutics, School of Pharmacy, Harbin Medical University, Harbin, P.R. China

**Keywords:** Lipid nanoparticles, pulmonary arterial hypertension, pulmonary delivery, pulmonary surfactant, resveratrol

## Abstract

In this study, we investigated the feasibility of dipalmitoylphosphatidylcholine-coated lipid nanoparticles (DPPC-LNs) as a carrier for preferential accumulation into lungs of Resveratrol (Res), a potentially promising drug for the treatment of pulmonary arterial hypertension (PAH). Res-loaded DPPC-LNs were prepared following a thin film hydration-ultrasonic dispersion technique using glyceryl monostearate as lipid core. DPPC can reduce the interactions between nanoparticles and pulmonary surfactant. The optimal formulation was prepared and characterized for physicochemical properties, storage stability and *in vitro* release profiles. The optimal formulation was evaluated for uptake by pulmonary arterial smooth muscle cells (PASMCs) using fluorescence microscopy. The efficacy of Res-loaded DPPC-LNs in reducing hyperplasia was tested in 5-HT induced proliferated PASMCs. The drug absorption profiles upon intratracheal administration were monitored in healthy rats. Optimized spherical DPPC-LNs – with mean size of 123.7 nm, zeta potential of –19.4 mV and entrapment efficiency of 94.40% – exhibited an 80% cumulative drug release over 48 h. Fluorescence microscopic study revealed an time-dependent enhancement of cellular uptake of Rh123-labeled DPPC-LNs by PASMCs. PASMC proliferation induced by 5-HT was significantly inhibited by Res-loaded DPPC-LNs. Optimized DPPC-LNs appeared to be safe when incubated with PASMCs. Besides, plasma and lung tissue data analysis indicated higher value of accumulation after intratracheal administration of Res-loaded DPPC-LNs in comparison with the intravenously dosed Res solution, indicating longer retention of Res in the lungs and their slower entry to the systemic blood circulation. DPPC-LNs could be a viable delivery system for site-specific treatment of PAH.

## 1. Introduction

Pulmonary arterial hypertension (PAH) is a cardiopulmonary progressive disease characterized by irreversible right ventricular failure, which ultimately leads to premature death. Common pathological features of PAH are vascular remodeling, manifested as medial thickening, pulmonary artery (PAs) muscle enhancement, and uncontrolled proliferation of pulmonary artery smooth muscle cells (PASMCs) (Epstein et al., 1994; Thompson & Lawrie, 2017). The structural changes of these small PAs will lead to gradual increase in arterial resistance and right ventricular dilatation (Yacoub & McLeod, 2018). Despite significant advances in PAH treatment over the past decade, the diagnosis of PAH is often accompanied by an extremely poor prognosis, even worse than many cancers (Hoeper et al., 2016). Existing treatments are mainly through the use of vasodilators such as prostaglandins, endothelin receptor antagonists and/or phosphodiesterase five inhibitors (Illaro Uranga et al., 2014; Humbert et al., 2014). Although these drugs relieve symptoms, they can only moderately improve survival (Pyne & Pyne, 2017). However, they cannot prevent excessive PASMCs proliferation and occlusive remodeling. This limited improvement of existing treatments highlights the urgent need to identify suitable targets to accommodate the remodeling of vascular dysfunction, which is the first step in developing effective anti-PAH drugs.

Currently, several studies have documented that Resveratrol (Res) possesses various biological properties, including antioxidant, anti-inflammatory, and anti-proliferative properties, and promotes cell differentiation and apoptosis (Csiszar et al., 2006; Ungvari et al., 2007; Fuggetta et al., 2016; Deus et al., 2017). Res, a silence information regulator 1 (SIRT1) activator, induces apoptosis MH7A human rheumatoid arthritis synovial cells in a SIRT1-dependent manner (Nakayama et al., 2012). SIRT1 attracts lots of interest in its cardiovascular protective role, which serves as a key regulator in vascular endothelial homeostasis by controlling angiogenesis, vascular tone, and endothelial dysfunction as well as by decreasing atherosclerosis (Zhou et al., 2011). Zhou et al. showed that Res upregulated the expression of the SIRT1 expression in PDGF-BB treated HPASMCs (Shuang et al., 2015). Furthermore, SIRT1 mediated the role of Res in regulating expression of cell cycle regulatory molecules and arresting PDGF-BB treated HPASMCs in G_0_/G_1_ phase, which contributed to the attenuation of pulmonary arterial remodeling and the alleviation of PAH in MCT-treated rats. Yu et al. showed that Res produced a beneficial effect partially by enhancing the activation of SIRT1, thus improving right ventricular systolic pressure and reducing right ventricular hypertrophy. SIRT1 activation increased PASMC apoptosis by inducing mPT dysfunction (Lei et al., 2017).

Although this molecule has therapeutic potential for PAH, Res presents pharmacokinetic limitations; for example, it is extensively metabolized after oral administration, resulting in low oral bioavailability. Additionally, a large portion of the dose is converted to conjugate sulfates, which is the limiting step in the systemic bioavailability of Res (Walle et al., 2004). The low aqueous solubility (log*P* of approximately 3.1) also favors the reduction of drug bioavailability, transforming its therapeutic and prophylactic potentials in a challenge. To circumvent this drawback, different strategies have been developed including the use of drug delivery systems such as cyclodextrins, liposomes, nano- and micro-particles (Amri et al., 2012; R. Neves et al., 2012; Martignoni et al., 2016). Inhalational route has been found to be rapid, safe, effective and cheap requiring lower dosage amount. This noninvasive and patient-friendly route is being increasingly tried for therapy of pulmonary diseases. In particular, DPPC-coated lipid nanoparticles (DPPC-LNs) which consist of a natural lipid-based solid core stabilized by a layer of pulmonary surfactant at the outer shell, exhibit several favorable characteristics as drug carrier including high biocompatibility and biodegradability, avoid macrophage uptake; low production cost, adequate physicochemical stability, protection of the incorporated active substance against degradation and modulation of its release (Scalia et al., 2014). Therefore, in this study, the main objective was to encapsulate Res in DPPC-LNs and to evaluate physicochemical properties, *in vitro* sustained release behaviors, cellular uptake and anti-proliferative effect and *in vivo* pharmacokinetics and lung retention of Res-loaded DPPC-LNs. The further objective was to test the hypothesis that Res-loaded DPPC-LNs via the pulmonary route were an effective carrier for providing sustained levels of Res in the lungs.

## 2. Materials and method

### 2.1. Materials

Phloretin (> 99%) as the internal standard and pure Res (> 99%) were purchased from Aladdin (Shanghai, China). Glyceryl monostearate (GMS) was kindly gifted from Gattefossé (Lyon, France). 1,2-dipalmitoyl-sn-glycero-3-phosphocholine (DPPC) was purchased from Nippon Fine Chemical (Japan). Polysorbate 80 was purchased from Fuyu chemical (Tianjing, China). Cell Counting Kit-8, Rhodamin 123 were both obtained from Meilunbio (Dalian, Liaoning, China). HPLC-grade methanol and acetonitrile were provided from Kermel (Tianjing, China). Purified water was supplied by Direct^®^Q^®^ water purification system (Millipore, Bedford, USA). Pulmonary arterial smooth muscle cells were supplied by the Department of Biopharmaceutical of Harbin Medical University (Daqing) and cell media was purchased from Hyclone (Logan, Utah, USA).

Male Sprague–Dawley (SD) rats (250 ± 15 g) were purchased from the Animal Center of the Second Affiliated Hospital of Harbin Medical University (Heilongjiang, China). The rats were only allowed free access to water before and during the experiment. The animals were used following the guidance of the Ethical Committee for Animal Experiments of Harbin Medical University.

### 2.2. Preparation of Res-DPPC-LNs

Res-loaded DPPC-coated lipid nanoparticles were prepared by a thin-film hydration-ultrasonic dispersion method. GMS was used to form lipid core. 1,2-dipalmitoyl-sn-glycero-3-phosphocholine (DPPC) as pulmonary surfactant was a shell-forming agent. Briefly, GMS (100 mg) and Res (50 mg) were dissolved in 10 ml ethanol in a round-bottom flask which was placed under vacuum in a water bath at 45 °C using an EL-131 Rotavapor (Buchi Laboratories AG, Postfach, Switzerland) to form a thin lipid film. For a complete removal of the organic solvent, the film was kept under vacuum for additional 1 h after film formation. Aqueous phases containing 1.0% *w*/*v* Polysorbate 80 and 1.25% *w*/*v* DPPC were simultaneously prepared at the same temperature. The dried lipid film was then rehydrated with aqueous phases containing 1.0% *w*/*v* Polysorbate 80 as the stabilizer. Crude emulsion thus obtained were sonicated for 2 min in ice water bath to prepare DPPC-LNs. We obtained purified particles by centrifuging the solution at 2,000 rpm for 10 min and washing the particles with Milli-Q water three times. The resulting solids were freeze-dried for 12 h and the powder of the DPPC-coated lipid nanoparticles was obtained.

### 2.3. Physicochemical characterization of Res-DPPC-LNs

The Res-DPPC-LNs were characterized for morphology, size, polydispersity index (PDI), zeta potential and entrapment efficiency (*EE*%). The morphology was studied in a transmission electron microscope (TEM) (Hitachi H-7650, Hitachi High Technologies America, Inc., Pleasanton, CA). The particle size and PDI of Res- DPPC-LNs was measured by dynamic light scattering (DLS) using a Zetasizer Nano ZS90 (Malvern Instruments Ltd., Worcestershire, UK), whereas the zeta potential was measured using the Zetasizer Nano ZS90 at 25 °C and a scattering angle of 17° by measuring the electrophoretic mobility with laser Doppler velocimetry. For characterization study, various parameters of optimal formulation were then measured three times.

For the determination of the *EE*%, the drug content was analyzed by reversed phase HPLC (Agilent 1260, USA). The un-encapsulated Res was separated from the DPPC-LNs by an ultrafiltration method (Hu et al., 2010) at the bottom of the Amicon Ultra^®^ tube (MWCO-100KD, Millipore Inc., Billerica, MA) and subjected to HPLC analysis to determine the content of Res. The total drug content in Res-DPPC-LNs was determined after lysis of lipid core with methanol. *EE*% could be calculated by the following equation:

$$\text{Encapsuation efficiency}\;(EE\%)=\frac{W_{t}-W_{u}}{W_{t}}\times 100\%$$

where *W_u_* is the drug untrapped in the DPPC-LNs and *W_t_* is the total drug in the DPPC-LNs.

### 2.4. *In vitro* release study

A modified dialysis method was used to evaluate the *in vitro* release of Res with or without loading in DPPC-LNs. Briefly, 1 ml of Res-DPPC-LNs or free Res with Res concentration of 5.0 mg/ml was placed in a regenerated cellulose dialysis bag (Spectra/Por^®^MWCO 8,000-14,000 Da, Spectrum Laboratories, Rancho Dominguez, CA, USA) against 200 ml of 0.5% SDS aqueous solution, which was maintained at 37 °C with continuous magnetic stirring at 100 rpm. At predetermined time intervals (0.5, 1, 2, 4, 6, 8, 10, 24, 36 and 48 h), 1 ml of solution in the receiving phase was taken for HPLC analysis of the released Res. After each 1 ml sample was collected for various time points and immediately replaced with the same volume of fresh media to maintain the total volume at 200 ml. The free Res was solubilized by the presence of 50% of PEG 400 in solution. All experiments were carried out in sextuplicate.

### 2.5. Stability of Res-DPPC-LNs

The stability of the formulation was evaluated by monitoring the size, zeta potential, PDI of the optimal formulation for over a month at a storage temperature of 4 °C and 25 °C. To determine if the drug is leaching out of the vesicles with time, the entrapment efficiency of Res-DPPC-LNs was also monitored periodically for a month. Briefly, the optimal formulation was prepared for characterization of size, zeta potential and entrapment efficiency as described above.

### 2.6. Cell viability study

The compatibility of the Res, DPPC-LNs and Res-DPPC-LNs were studied by determining the viability of PASMCs using the Cell Counting Kit-8 (CCK-8) (Meilun Biotechnology, Dalian, China) according to the manufacturer’s protocol. Briefly, 50,000 PASMCs were seeded on 96 wells plates in DMEM with 10% FBS and kept overnight for cells to attach. Next day, plain Res, DPPC-LNs and Res-DPPC-LNs containing 25, 50, 100 or 200 μM Res was added to 96-well plates and incubated for 24 h at 37 °C. The culture media was then replaced with 100 μl of fresh media containing 10% CCK-8 and incubated for another 2 h. Finally, the viable cells were detected using the Cell Counting Kit-8, where the absorbance for each sample was assessed at 450 nm using a microplate reader (TECAN, Salzburg, Austria). The results were expressed as mean values ± standard deviation (SD) of six measurements. The percent of viable cells were calculated from the following equation:

$$\mathrm{Cell}\;\mathrm{Viability}\;(\%)=\frac{A_{S}-A_{b}}{A_{c}-A_{b}}\times 100\%$$

where *A_s_* is absorbance of experimental wells (medium containing cells, CCK-8, drug), *A_c_* is absorbance of control well (medium containing cells, CCK-8), and *A_b_* is the absorbance of blank wells (medium without cells and drug, CCK-8).

### 2.7. Cellular uptake study

Particle uptake by rat PASMCs was evaluated by incubating the particles with rat PASMCs. For cellular uptake study, the uptake of DPPC-LNs by rat PASMCs was tested at three time points (1, 2 and 4 h) using a fluorescence microscope. In short, rat PASMCs were cultured in 25 cm^2^ flask in 10% FBS, 1% penicillin/streptomycin and glutamine containing DMEM at 37 °C in 5% CO_2_ atmosphere. PASMCs (10,000) were seeded on 12 wells plates and kept overnight to attach on the surface of well. The cells were then incubated with fluorescent-labeled (rhodamine 123) DPPC-LNs for 1, 2 and 4 h. After washing three times with PBS, the cells were fixed with 4% freshly prepared paraformaldehyde solution and stained with DAPI (4′,6-diamidino-2-Phenylindole, Dihydrochloride). The cells were then viewed under a fluorescent microscope (IX-71, Olympus, Center Valley, PA).

### 2.8. Cell proliferation assay

The effect of Res-loaded DPPC-LNs in attenuating proliferation of PASMCs was evaluated by CCK-8 assay. Cells were seeded on 96 wells plate and kept overnight to get attached on the well surface. Next day, the cells were incubated with 25, 50 and 100 μM plain Res or Res- DPPC-LNs containing equivalent amount of Res for 24 h. The incubation was performed to evaluate the relative efficacy in inhibiting cell proliferation. Cells were then stimulated with 2 μM 5-HT for 24 h followed by incubation with fresh media containing 10% CCK-8 at 37 °C for 2 h. Finally, the viable cells were detected as described above.

### 2.9. *In vivo* studies

#### 2.9.1. Animals

Male SD rats with an average weight of 250 ± 15 g were used for this study (*n* = 24). The animal experiments were carried out in accordance with approved ethical protocols of the Animal Care Committee of Harbin Medical University, Harbin, Heilongjiang, China (Ethics approval number: SCXK (Hei) 2013-002). Rats were maintained in cages with free access to water, but deprived of food overnight before the experiments with a 12h light/darkness cycle and ambient temperature.

#### 2.9.2. Experimental procedure

Before intratracheal inhalation of the optimum Res-DPPC-LNs aerosol, each rat was anesthetized by intraperitoneal injection of a mixture of ketamine (90 mg/kg body weight) and xylazine (9 mg/kg body weight). The Res-DPPC-LNs suspensions were obtained by dispersing Res-DPPC-LNs in water. After exposing the trachea using a laryngoscope, Microsprayer^®^ for rat (Model IA-1B; Penn Century Inc., USA) was used for intratracheal administration and the dose of the drug was 10 mg/kg. After intratracheal dosing, the animals were held in an upright position for 1 min to ensure deposition of the dose following the removal of the delivery device. For intravenous administration (tail vein injection), free Res solution was prepared by dissolving Res in 50% PEG 400 aqueous saline (0.9% NaCl) solution, and then the volume of free Res solution equal to 10 mg/kg for each rat was accurately measured.

For plasma pharmacokinetic studies, the rats were randomly divided in two groups of six animals each. Following the drug administration as described above, blood samples (0.5 ml) were taken from orbital vein at 5, 15, 30, 45, 60, 90, 120, 150, 180, 240, 480 and 720 min. For plasma separation, samples were centrifuged at 5510× *g* for 10 min, and then the collected plasma was stored frozen (–20 °C) until future use.

The other group, with 72 rats, was used for the assessment of Res concentration within the lung tissue. Twelve rats (*n* = 6 for each time point) were sacrificed at various time points (5, 30, 60, 120, 240, and 720 min) after intratracheal inhalation of the optimum Res-DPPC-LNs or intravenous dosing of free Res solution. At each sampling time point, lung tissues were removed, rinsed twice with normal saline (0.9% *w*/*v*), and wiped with a filter paper. After accurate weighing of the specimens, the samples were stored frozen (–20 °C) until extraction procedure.

#### 2.9.3. Sample preparation and analysis

The sample preparation method was similar for both the plasma and lung samples, except that the lung tissue was homogenized with three volume of normal saline (0.9% *w*/*v*) (Jingxin, F6/10, high-speed homogenizer, China) before the procedure. 10 μl of Phloretin solution (35 μg/ml) as internal standard was added to 100 μl of lung or plasma sample and vortex-mixed for 2 min. Afterward, 1.5 ml of mixture of methyl tert-butyl ether and dichloromethane (*v*:* v* = 2: 1) as extraction solvent was added to each sample. Finally, the resultant mixture was vortex-mixed for 5 min and centrifuged (15,300× *g*, 10 min) at 4 °C (Eppendorf, 5430 R, high speed refrigerated microcentrifuge, Germany). After centrifugation, the supernatant layer was separated and dried under a gentle stream of N_2_ at room temperature (L-119A, Termovap Sample Concentrator, China). The dried residual was then reconstituted in 100 μl of the mobile phase, vortex-mixed for 5 min, and centrifuged for 10 min at 14550× *g*. Finally, the supernatant was injected to HPLC for analysis.

Res concentration in the plasma and lung samples, was determined by a previously reported HPLC method (Musazzi et al., 2014) with some modifications. The separation was performed on a Diamonsil C_18_ column (250 × 4.6 mm, 5 μm) (ODS, Dikma, China) at 30 °C and the injection volume was 10 μl. The isocratic mobile phase was a mixture of acetonitrile and water in a ratio of 38 : 62. The flow rate was 1 ml/min and the analysis was carried out by a Diode array UV detector (Agilent, USA) at 305 nm. The retention times for Phloretin as internal standard and Res were 5.4 and 7.9 min, respectively.

### 2.10. Data analysis

The pharmacokinetic parameters were calculated by DAS (The drug and statistics software, version 2.0). Statistic analysis was carried out by the unpaired student’s t-test using SPSS statistic program (version 19.0), with a statistical significance level of *p* < 0.05. All data were expressed as the means ± SD.

## 3. Results and discussion

### 3.1. Physical characterization

**Figure 1.**
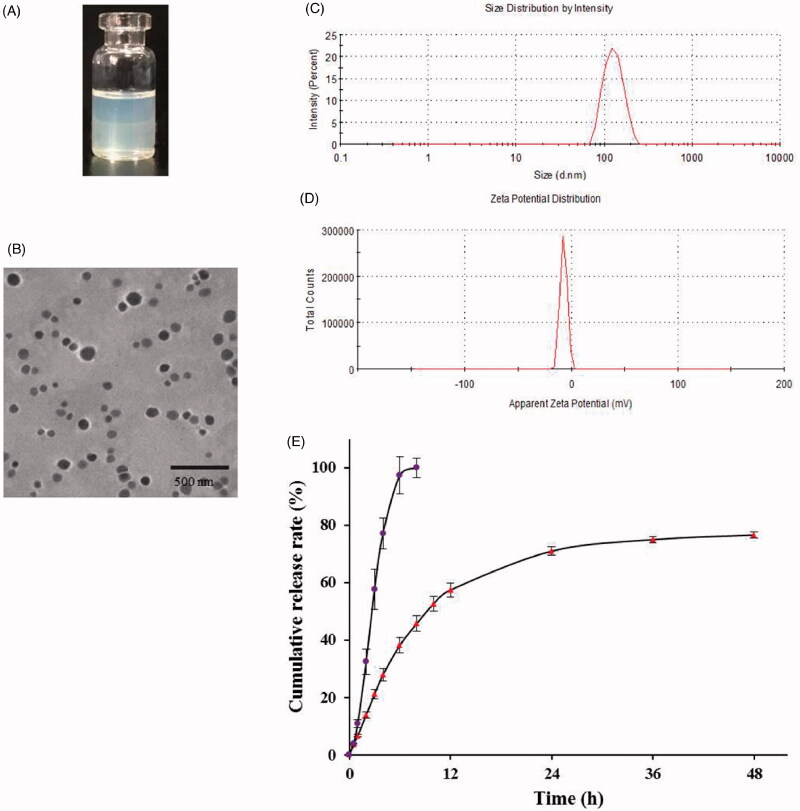
Characterization of Res-DPPC-LNs. (A) the appearance of Res-DPPC-LNs suspensions, (B) transmission electron microscopic image, (C) size distribution, (D) zeta potential, (E) *In vitro* release profiles of plain Res and Res-DPPC-LNs suspensions in pH 7.4 PBS at 37 °C (Data represent mean ± SD, *n* = 6). () plain Res, () Res-DPPC-LNs.

The appearance of Res-DPPC-LNs dispersed in water is shown in Figure 1(A). The TEM image (Figure 1(B)) of the optimized Res-DPPC-LNs suspension indicated a spherical shape and no adhesion. The white shell around particles was present in the surface of the DPPC-LNs which represented DPPC forming a pulmonary surfactant shell. Surface coating with DPPC makes particles inert to pulmonary surfactant layer in lung and hence reduces the chances of their recognition by macrophages (Guagliardo et al., 2018). The particle size of DPPC-LNs with or without Res was determined to be 123.7 ± 16.43 nm and 67.31 ± 30.10 nm, respectively, with relatively narrow size distribution indicated by PDI values of 0.082 and 0.151 (Figure 1(C)). Entrapment of Res appeared to contribute to the size of the DPPC-LNs based on the observation that DPPC-LNs containing Res were larger than plain DPPC-LNs. Another important parameter, i.e., poly dispersity index (PDI) that is a dimensionless number that measures the breadth of size distribution, which indicates whether a colloidal system is monodisperse or heterodisperse. PDI values vary from 0.05 to 1.0; a PDI of 1.0 indicates a very broad size distribution. PDI of the DPPC-LNs were less than 0.2, which indicates a monodispersed colloidal system. The zeta potentials (Figure 1(D)) of Res-loaded DPPC-LNs were between –19.4 ± 3.31 mV, suggesting that the electrostatic repulsion might prevent the particle aggregation and increase the stability of the dispersions.

*EE*% is one of primarily important characteristics for evaluating the quality of DPPC-LNs. After separating the unentrapped Res using a previously described ultrafiltration method, *EE*% was determined to be 94.40 ± 0.65%. The high *EE*% indicated that GMS-based DPPC-LNs efficiently entrap Res.

### 3.2. *In vitro* release of Res-DPPC-LNs

The *in vitro* release profile of Res-DPPC-LNs and free Res solution is shown in Figure 1(E). It can be seen that the free Res solution led to a relatively rapid release with more than 97% of drug released from the dialysis bag in 6 h. When encapsulated into DPPC-LNs, Res was released in a sustained manner over 48 h with less than 40% of initial burst within the first 5 h. Such significantly sustained release was likely attributed to the slower diffusion of Res from DPPC-LNs rather than the penetration of drug molecules across dialysis membrane, and the *in vitro* release data also suggested that the present DPPC-LNs formulations were potentially useful to control the release of Res.

To determine the mechanism of drug release, drug release data (both burst and sustained release) were fitted with kinetic models and the results obtained from data fitting are summarized in Table 1. The Makoid–Banakar release model mainly examines the diffusion index *n*, where *n* < 0.45 indicates that the release model is a diffusive type, *n* > 0.89 represents an erosive type, and 0.45 < *n* < 0.89 represents a combination of erosion and diffusion. Based on the selection criteria: *R*^2^, it can be seen that the release data fit well to the Makoid–Banakar model, and *n* = 0.708, indicating that the release behavior of Res from DPPC-LNs is combination of erosion and diffusion. It is likely that the drug is encapsulated both within the core of DPPC-LNs (resulting in slow, sustained release) and within the shell or surface of DPPC-LNs (resulting in fast “burst” release).

**Table 1.** Results of model fitting of release curves of Res-DPPC-LNs in PBS.

Model	Equation	*R*^2^	*n*
Zero-order	*F*=*k*_0_*t*	0.7992	**/**
First-order	*F* = 100[1-$\;e^{-k1t}$]	0.9726	**/**
Higuchi	*F*=*k*_H_$t^{0.5}$	0.9167	**/**
Makoid-Banakar	*F*=*k*_MB_$t^{n}e^{-\mathrm{kt}}$	0.9927	0.708

### 3.3. Stability of Res-DPPC-LNs

**Figure 2.**
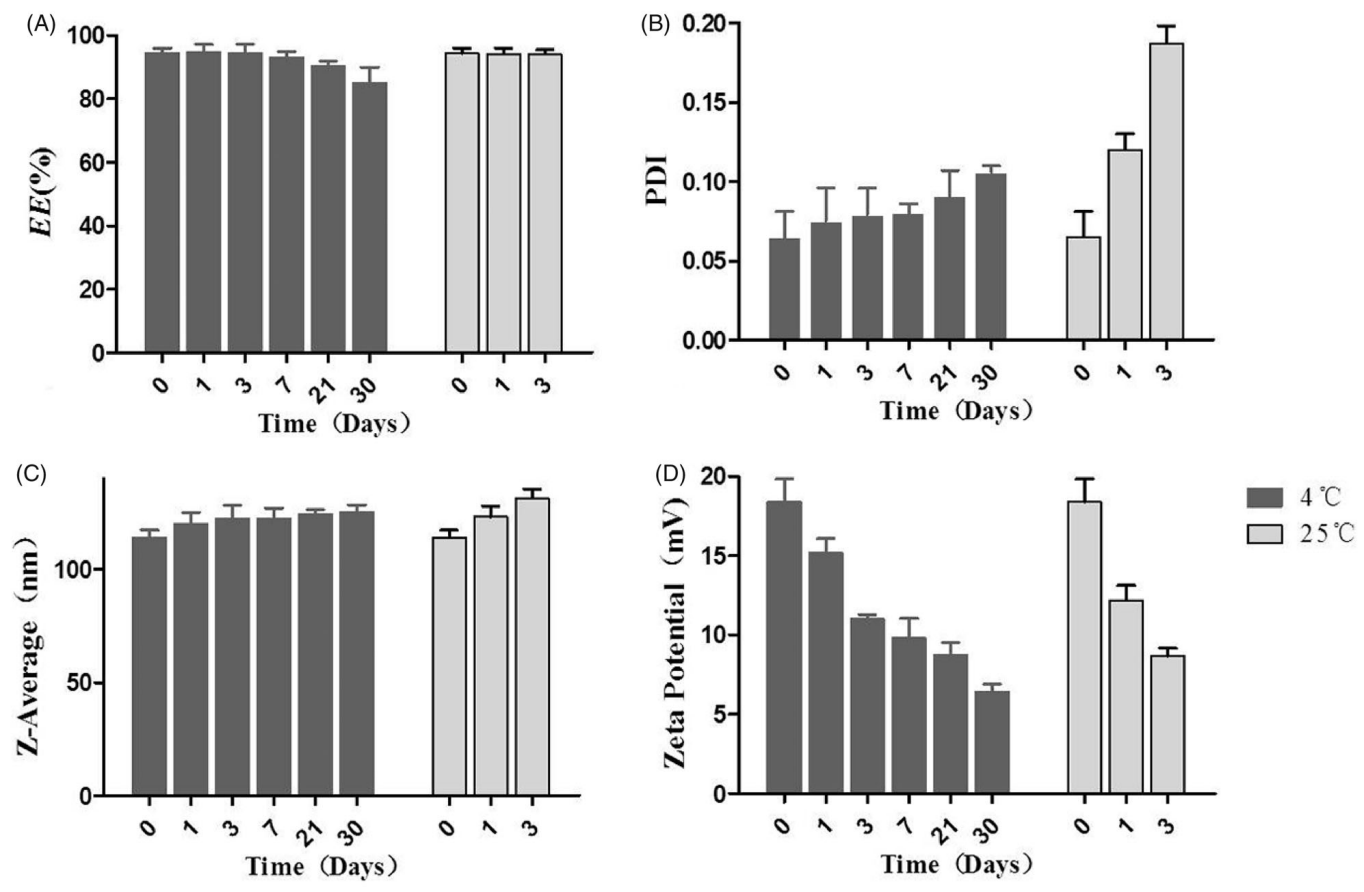
Physicochemical stability of Res-DPPC-LNs: (A) entrapment efficiency, (B) PDI, (C) size and (D) zeta potential. Data represent mean ± SD, *n* = 3.

The stability of Res-DPPC-LNs at two different storage temperatures, 4 °C and 25 °C, was evaluated in terms of *EE*%, size, PDI and zeta potential. Res-DPPC-LNs were not stable even for 7 days at 25 °C. However, Res-DPPC-LNs were stable for more than 30 days at 4 °C (Figure 2). Res-DPPC-LNs stored at 4 °C did not show significant change in the amount of entrapped drug; but Res-DPPC-LNs dispersion has precipitated when stored at 25 °C for 7 days. Within 7 days, Res-DPPC-LNs were aggregated as evident by an increase in size of the Res-DPPC-LNs along with formation of sediments at 25 °C. The PDI increased slightly when stored at 4 °C for 30 days, but the PDI increased significantly after 3 days storage at 25 °C, suggesting a change in formulation stability. Furthermore, significant change in the zeta potential was observed during the storage period, indicates that the stability of Res-DPPC-LNs will gradually decrease after being dispersed in water, especially at 25 °C.

### 3.4. Cell viability study

**Figure 3.**
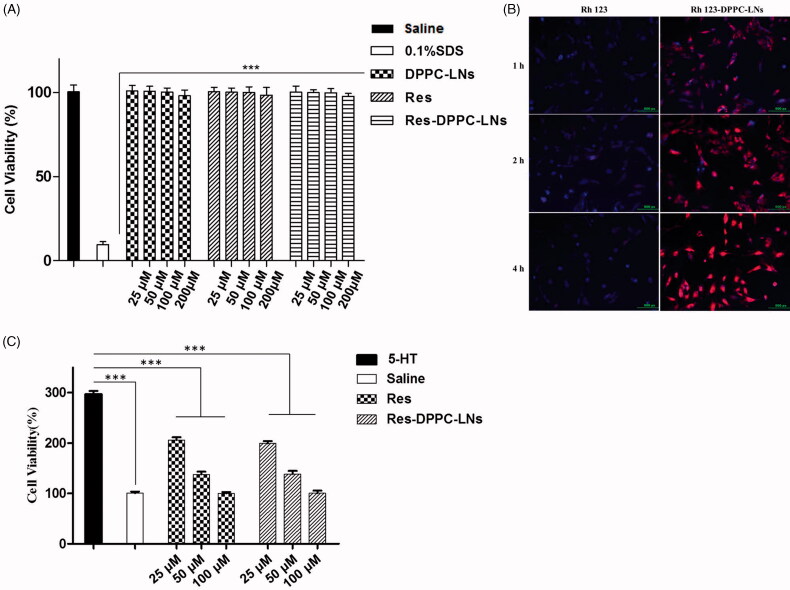
(A) Viability of rat PASMCs upon incubation with formulations or individual components of formulation. (mean ± SD, *n* = 6), (B) Representative fluorescence microscopic images showing the uptake of Free Rh123 and Rh123-DPPC-LNs by pulmonary arterial smooth muscle cells. Blue color represents cell nucleus stained with DAPI. Red color represents Rh123, (C) Effect of Plain Res and Res-DPPC-LNs on 5-HT induced PASMC proliferation (mean ± SD, *n* = 6). *** *P* < 0.001.

Despite that all the excipients reported in this study are considered GRAS, their toxicity may differ based on their concentration or route of administration. Therefore, their cytotoxicity against PASMCs was assessed by CCK-8 assay and is presented in Figure 3(A). PASMCs were incubated with serial dilutions of the DPPC-LNs and active formulations (25, 50, 100, 200 μM). The viability of PASMCs was unaffected with the increasing concentration of plain Res (from 25 μM to 200 μM) or equivalent concentration of DPPC-LNs and Res-DPPC-LNs.

### 3.5. Uptake of DPPC-LNs by PASMCs

The site of action of Res is the PASMCs and the target enzyme, SIRT1, is expressed in this cell. When PASMCs were incubated for 1, 2 and 4 h with fluorescent DPPC-LNs, Rh123-labeled

DPPC-LNs were taken up by the cells with a time-dependent increase (Figure 3(B)). Similarly, PASMCs treated with free (non-loaded) Rh123 also showed very little fluorescence indicating that the DPPC-LNs were required for Rh123 uptake. It is clear from the results that cell uptake of DPPC-LNs was mediated by endocytosis. Similar results have been previously reported by other researchers (Martins et al., 2012).

### 3.6. Effect of Res-DPPC-LNs on cell proliferation

A characteristic feature of PAH is the proliferation of PASMCs (Mclaughlin & McGoon, 2006). Thus, the effect of Res-DPPC-LNs in attenuating cell proliferation was tested in 5-HT induced PASMCs. Treatment with plain Res and Res-DPPC-LNs attenuates 5-HT induced proliferation of PASMCs (Figure 3(C)). When Res-DPPC-LNs (from 25 μM to 200 μM) were incubated with PASMCs, a remarkable reduction of cell proliferation was observed which was similar to that of plain Res.

### 3.7. *In vivo* studies

The concentration of Res in plasma and lungs was determined by a validated RP-HPLC method. In brief, the calibration curves of Res in plasma and lungs were found to be linear over the concentration range of 0.10–16.00 μg/ml and 0.1–2.00 μg/ml, the corresponding regression equations were Y = 1.0086X + 0.0397, *r*^2^ = 0.9991 and Y = 1.5722X–0.1127, *r*^2^ = 0.9956, where X is Res concentration (μg/ml) and Y is peak area ratio of Res to internal standard. Besides, at both analysis, the accuracy was in the range of 90–110% and the recovery was > 80%.

**Figure 4.**
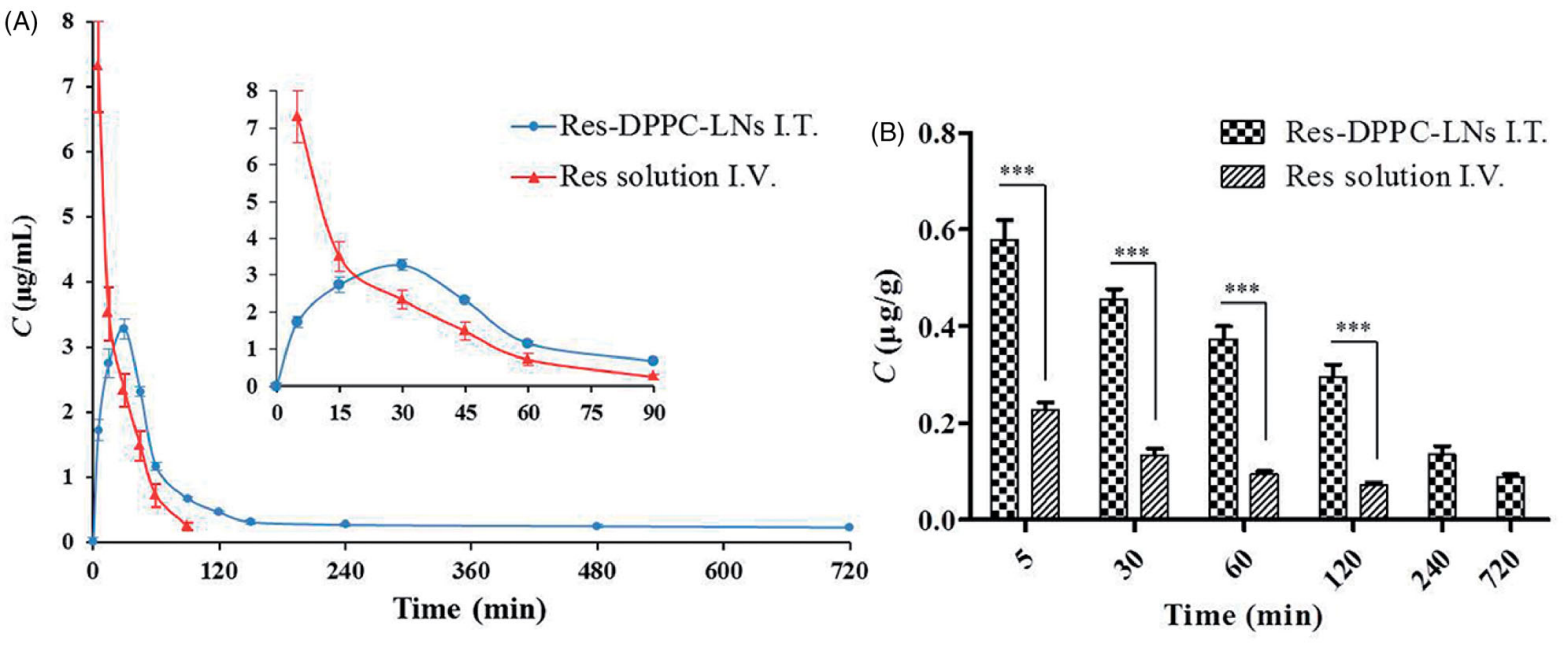
(A) *In vivo* absorption profile of Res following intratracheal inhalation of the Res-DPPC-LNs and intravenous administration of the Res solution to rats. (dose = 10 mg/kg, mean ± SD, *n* = 6), (B) The Res lung concentration-time profiles following intratracheal inhalation of the Res-DPPC-LNs and intravenous administration of the Res solution to rats. (dose = 10 mg/kg, mean ± SD, *n* = 6).

The plasma pharmacokinetic parameters and the plasma and lung concentration-time profiles of Res following intratracheal inhalation of the optimum Res-DPPC-LNs and intravenous administration of the free Res solution to rats are shown in Table 2 and Figure 4(A), respectively. There is some evidence that more retention in the lungs and higher local drug concentration are associated with better efficacy in the treatment of PAH with less systemic side effects (Feng et al., 2011; Sun et al., 2012). Ventavis^®^ and Tyvaso^®^ are the examples of approved inhalation products for the treatment of PAH, highlighting the efficacy of local treatment. From the results, compared with intravenous administration of the free Res solution, intratracheal administration of the optimum Res-DPPC-LNs resulted in significant increase in the pharmacokinetic parameters of *AUC*_0–∞_, *AUMC*_0–∞_, and *MRT* (about 1.9, 23.8, and 12.2-fold, respectively) (*P* ˂ 0.001). The higher *AUC* for the inhalation product, which is indicative of enhancement in Res bioavailability can be due to some reasons, including improvement of solubility and dissolution rate of Res by nanosizing, the lack of hepatic first-pass elimination, and lower enzymatic activity in the lungs. Furthermore, absorption of Res molecules across the very thin alveolar-capillary barrier (600–800 nm) to the systemic circulation can be facilitated by the presence of DPPC, as a component of lung surfactant, in the optimum Res-DPPC-LNs formulation, allowing the inertia of the nanoparticles to the lung surfactant layer, which can be better transported to action site through the mammalian lung surfactant consisting of 85–90% phospholipids and 8–10% of fatty acids, cholesterols, and specific proteins (Mahmud & Discher, 2011; Dwivedi et al., 2014). The half-life (*t*_1/2_) of free Res solution (0.30 ± 0.01 h) was significantly lower than the inhaled one (4.39 ± 1.12 h), *P* < 0.001, which are attributed to drugs sustained release from nanoparticle following administration of the drug in rat.

**Table 2.** The plasma pharmacokinetic parameters of Res following intratracheal inhalation of the Res-DPPC-LNs and intravenous administration of the Res solution to rats.

Parameters	Intratracheal administration Res-DPPC-LNs	Intravenous administration plain Res	*p* Value
*MRT*(h)	5.01 ± 0.91	0.41 ± 0.02	0.00
*t*_max_(h)	0.50 ± 0.00	–	–
*t*_1/2_(h)	7.73 ± 2.29	0.32 ± 0.02	0.00
*AUC*_0→t_(μg/mL·h)	5.08 ± 0.44	3.35 ± 0.43	0.00
*AUC*_0→∞_(μg/mL·h)	6.44 ± 0.77	3.39 ± 0.44	0.00
*AUMC*_0→∞_(μg/mL·h^2^)	33.26 ± 10.17	1.40 ± 0.25	0.00
*Cl/F* (L/h)	1.57 ± 0.18	3.00 ± 0.39	0.00
*V*_ss_*/F* (mL)	17.17 ± 4.00	1.32 ± 0.09	0.00
*C*_max_ (mg/mL)	3.28 ± 0.22	–	–

(dose = 10 mg/kg, mean ± SD, *n* = 6).

Assessment of lung tissue showed that 5 min after intratracheal administration, Res concentration in the lungs was 0.5796 ± 0.0979 μg/g, which was about 2.5-fold of that of intravenous administration of the free Res solution (0.2280 ± 0.0371 μg/g), and this ratio increased to about 4-fold at 120 min after administration. Rapid onset of therapeutic action is another advantage of inhalation drug delivery, which is due to the fact that this route of administration allows bypassing the time in the distribution from the intravenous to lung, and thus achieving maximum local drug concentration from the beginning of aspiration, whereas, following intravenous administration, Res concentration in the lungs decreased rapidly within 120 min. Inhaled Res-DPPC-LNs showed a sustained drug release over 720 min (Figure 4(B)) Since the shell of the inhaled particles was pulmonary surfactant, thus it is possible that a fraction of the particles was not recognized by the phagocytic cells and substantially not cleared from the lungs before reaching the desired action site. This potentially explains the drug concentration in the lungs still detected after intratracheal administration instead of intravenous administration at 720 min, which could be better for the treatment of PAH. Previously, Hu et al. reported that the spray dried nanocrystals of curcumin acetate, with long retention in the lungs, can be an effective treatment in a rat model of monocrotaline-induced PAH (Hu et al., 2017).

As far as we know, no report has been published about the pharmacokinetic characteristics of Res after intratracheal inhalation, and thus this study suggests a new strategy for improving the effectiveness of Res in the treatment of PAH.

## 4. Conclusion

This study shows the feasibility of DPPC-coated lipid nanoparticles as carriers for pulmonary delivery of Res, a potential anti-PAH drug. Indeed, favorable physicochemical properties, *in vitro* sustained release behaviors, enhanced cellular uptake and significant anti-proliferative effect proved that the applicability of DPPC-coated lipid nanoparticles for inhalational delivery. In addition, the results of the *in vivo* evaluation indicated that inhaling the DPPC-coated lipid nanoparticles provides the advantages of rapidly producing treating effect, a higher local drug concentration, a longer retention of drug in lungs and, in consequence, an effective drug delivery to the lungs compared to intravenous administration of Res. However, the effect of the formulations should be further evaluated by toxicological, pharmacological, and clinical studies.
